# Dry biocleaning of artwork: an innovative methodology for Cultural Heritage recovery?

**DOI:** 10.15698/mic2021.05.748

**Published:** 2021-04-15

**Authors:** Giancarlo Ranalli, Pilar Bosch-Roig, Simone Crudele, Laura Rampazzi, Cristina Corti, Elisabetta Zanardini

**Affiliations:** 1 Department of Bioscience and Territory, University of Molise, Pesche, Italy.; 2 Department of Conservation and Restoration of Cultural Heritage, Instituto de Restauration de Patrimonio, Universitat Politècnica de València, Valencia, Spain.; 3 Department of Human Sciences, Innovation and Territory, Università degli Studi dell'Insubria, Como, Italy.; 4 The Institute of Heritage Science, National Research Council of Italy, Milan, Italy.; 5 Department of Science and High Technology, Università degli Studi dell'Insubria, Como, Italy.

**Keywords:** dry biocleaning, stonework, yeast, cultural heritage, on-site biotreatment

## Abstract

An innovative methodology is proposed, based on applied biotechnology to the recovery of altered stonework: the “*dry biocleaning*”, which envisages the use of dehydrated microbial cells without the use of free water or gel-based matrices. This methodology can be particularly useful for the recovery of highly-ornamented stoneworks, which cannot be treated using the conventional cleaning techniques. The experimental plan included initial laboratory tests on Carrara marble samples, inoculated with dehydrated Saccharomyces cerevisiae yeast cells, followed by on-site tests performed on “*Quattro Fontane*” (*The Four Fountains*), a travertine monumental complex in Rome (Italy), on altered highly ornamented areas of about 1,000 cm^2^. The mechanism is based on the spontaneous re-hydration process due to the environmental humidity and on the metabolic fermentative activity of the yeast cells. Evaluation by physical-chemical analyses, after 18 hours of the biocleaning, confirmed a better removal of salts and pollutants, compared to both nebulization treatment and control tests (without cells). The new proposed on-site *dry biocleaning* technique, adopting viable yeast cells, represents a promising method that can be further investigated and optimized for recovering specific altered Cultural Heritage stoneworks.

## INTRODUCTION

Microorganisms can represent a double-edged sword for Cultural Heritage (CH); negatively, they can have serious detrimental effects on artistic materials [1–13].

Microorganisms become virtuous when used for the removal of harmful compounds from CH [14–27]. In the last two decades, many applied studies have shown that biocleaning of altered CH surfaces has some advantages over the traditional cleaning methods; indeed, chemical treatments are not always sufficiently selective consequently damaging stone surfaces [28–31].

However, traditional cleaning methods, based on chemical and mechanical treatments, are still preferred, as microorganisms are generally linked to their deterioration agents [21]. The drawbacks of the traditional cleaning procedures, chemical, mechanical and laser systems, should be shown. In this context, in order to inhibit or eradicate biological colonization, chemical and physical methods are used, separately or in combination, and include active compounds generally toxic and not degradable, which can be persistent in the environment and have a negative impact on the treated areas [31–33].

Nevertheless, it is worth to mention the use of conventional miniaturized devices suitable to perform *in situ* investigation, diagnosis and monitoring of the conservation status of CH artworks which include new portable and movable smart sensors [34].

Biocleaning methods are less-invasive compared to the chemical treatments and represent an environmentally-friendly alternative as microorganisms act in the same way as they would do in their natural environments [9, 35, 36]. To date selected bacteria have been used for the removal of sulfates, nitrates and organic matter from stone [37–43]. Bacteria such as *Pseudomonas stutzeri, Pseudomonas aeruginosa, Desulfovibrio vulgaris* and *Desulfovibrio desulfuricans* are able to remove undesirable salts such as nitrates and sulfates from marble, brick and calcareous stone and wall paintings, both in the laboratory and on-site [44–51]. The effectiveness of the cleaning methods is traditionally verified by chemical analysis of the treated surface [52, 53]. In many cases, the use of an inert delivery system has been more successful than the direct application of microorganisms alone; in fact, the delivery system can provide the right amount of water that is essential for cell survival and their metabolic activity on stone surface [54]. Recent studies and reviews on the CH biocleaning describe the characteristics and properties of different delivery systems comparing them in terms of ease of application and related costs [9, 20, 55–57]. Several factors can influence the results of the biocleaning treatments on CH artwork: the type of materials (paper, stone, wood, textiles, metal, glass, etc.), the type of alterations and, for stonework and frescoes, the mineral matrices [58]. No less important are the long effects of the biocleaning treatments when they are performed outdoors under variable meteorological and climatic conditions (such as temperature, relative humidity and condensation phenomena).

From a different point of view, water content applied to CH surfaces during biocleaning treatments can be a problem in some cases (for example causing leaching or salt solubilization), since it can affect the state of the CH materials. For this reason, a dry system has been considered in this study. Moreover, the works of art to be treated are very often fragile and delicate when exposed to water because of the state of the material and/or environmental stresses.

In our study, the City of Rome Superintendency recommended the use of a cleaning method that did not require the addition of large amounts of water to avoid salt solubilization on the artwork to be treated and the risks of metal oxidation processes leading to undesirable stains on the stone surfaces.

Then our investigation started from the following idea: a treatment performed without the addition of free water and the use of dried yeasts, which can become active after the spontaneous re-hydration in outdoor environment, directly on the altered CH surface.

For the first time, this paper reports the use of the metabolism of yeast cells to biorestore monuments, adopting a dry biocleaning process. This method can be described as a “*Pandoro effect*” referring to a typical Italian Christmas cake in which fine powdered sugar is added to the surface just before consumption, similarly to the addition on artwork surface of powdered dry yeasts as reported in this case-study. Dry conditions have been shown to be a good cleaning option in different fields, for example for textiles and hair. *Saccharomyces cerevisiae* is the best-known yeast, involved in winemaking, baking, and brewing since ancient times [59, 60]. It is a eukaryotic unicellular organism belonging to the *Saccharomycetaceae* family, in the *Ascomycota* phylum. It reproduces by budding and, like all yeast strains, *S. cerevisiae* can grow aerobically or anaerobically on various carbohydrates, such as glucose, galactose, fructose and maltose, the last two of which have been shown to be the best at fermenting sugars. Yeasts growth rate varies according to specific nutritional conditions such as the availability of sources of nitrogen (from ammonia and urea, but not nitrates), amino acids and small peptides. Furthermore, yeasts require phosphorus and sulfur, which can be assimilated as the amino acids methionine and cysteine, and some metals, including magnesium, iron, calcium and zinc, which are required for a good growth.

The aims of the present study were to assess:

- the performance of selected dried yeast cells in the biocleaning of salts, deposits and atmospheric pollutants altering a characteristic limestone (travertine), on the on-site external surfaces of *Quattro Fontane*, Rome (Italy);
- the set-up and optimization of an advanced dry biocleaning protocol for altered surfaces;
- the effectiveness in terms of physical-chemical and biological analyses;
- the potential risks for the works of art over the short and medium term; and
- the impact of the innovative biocleaning process with a view to on-site CH recovery.

## RESULTS

### Lab scale dry biocleaning

At the laboratory scale, preliminary dry biocleaning tests were carried out on two different specimens: (sample 1) an ordinary flat carbonate stone and (sample 2) a *curved* fine CH marble fragment (horse-hoof, XIV century, OpaPisa, Italy), reported in **Table 1**.

**TABLE 1.** Main physical-chemical and biological data on the surfaces of both the common carbonate stone (sample 1) and CH marble (sample 2) fragments, before (T0) and after (T48 hours) the dry biocleaning (DB) process, at the laboratory scale (three replicates, mean ± SD).

**Stone**		**Sample 1 Common carbonate stone**		**Sample 2 CH marble**			
				**DB vs. C**		**C vs. fresh cut**	
**Parameters/Time (hours)**		0-48		0-48		0-48	
**Physical**	Color changes						
	ΔL*	9.04		49.30		4.10	
	Δa*	7.70		−1.30		−0.73	
	Δb*	1.88		0.65		−1.45	
	ΔE*_ab_	12.60		49.80		4.40	
	Water absorption (g)	0.3±0.1	0.4±0.1	0.8±0.3	1.4±0.5	0.8±0.2	0.9±0.3
**Chemical**	pH surface	6.9	6.1	6.9	6.3	6.9	6.9
	Alcohol (%)	0.0	0.15	0.0	0.10	0.0	0.0
**Biological**	Bacteria (CFU cm^−2^)	3±1	1±1	2±2	2±1	2±2	2±1
	Fungi (CFU cm^−2^)	2±1	1±1	1±1	2±2	1±1	3±2
	Yeasts (CFU cm^−2^)	0	18±2	0	25±4	0	0
	ATP (pg cm^−2^)	13.3±2	2,100±15	7.2±5	3,700±22	5.3±3	7.3±2
	Number of enzymes	2	11	2	9	2	2

**Figure 1:**
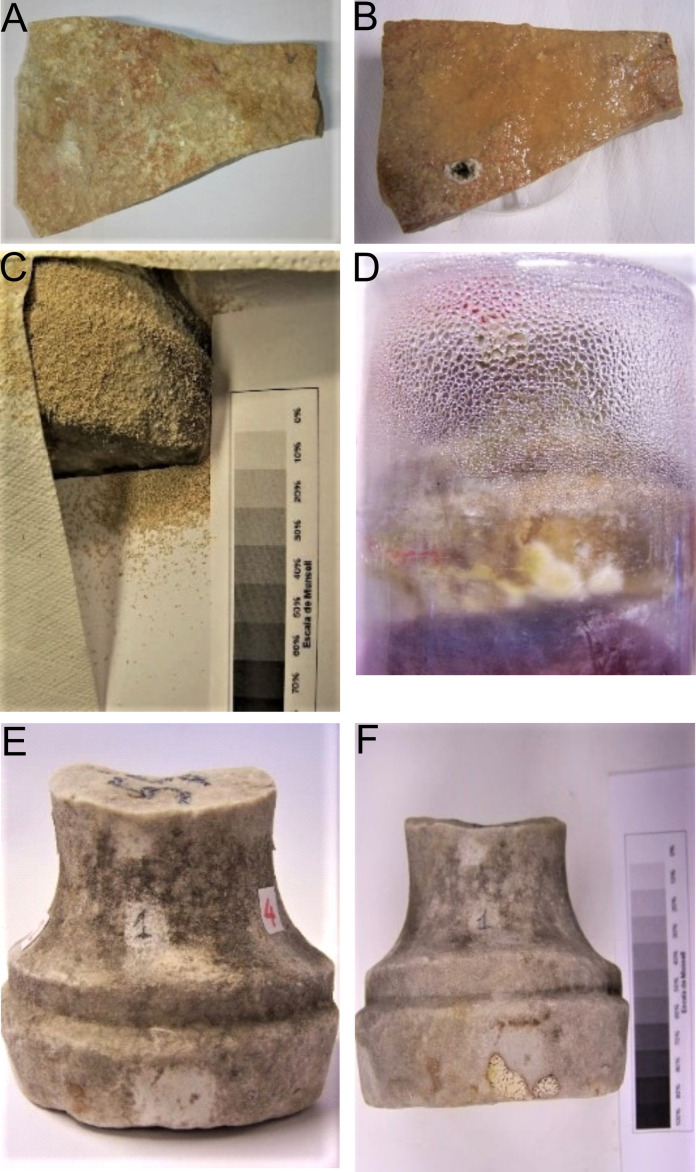
FIGURE 1: Laboratory scale dry biocleaning on two specimens. (Top) sample 1 (A–B), a common flat stone (carbonate stone) before and after yeast-sucrose addition; (middle) sample 2 (C-D), a CH marble fragment, after yeast-sucrose addition and detail of incubation in the Gas-Pak jar with condensation phenomenon; (bottom) sample 2 (E-F), the CH marble surface before and after the dry biocleaning process.

Dried yeast cells, added to a carbonaceous material in a highly purified form (micronised sucrose) in order to favor their adhesion and hydration by high hygroscopicity, were adopted. The subsequent incubation within the GasPak system, in the presence of solely internal humidity, confirmed abundant microbial metabolism of *S. cerevisiae* cells on the surfaces of both common carbonate stone and CH marble as shown in **Figure 1**.

In detail, the lab scale dry-biocleaning was optimized as follows:

-) characterization of the main parameters, at time zero (see section “Materials and Methods”);
-) cooling at 4 °C for 1 hour;
-) manual application of dried yeast-carbon source onto stone fragment surfaces;
-) incubation of inoculated stone fragments in the GasPak system at 28°C for 48 hours;
-) checking for spontaneous re-hydration process and condensation phenomena on stone fragment surfaces;
-) stone fragment recovery, surface gently sponged clean and dried at room temperature;
-) final characterization as in point 1.

At the laboratory scale, the surface stone fragments sample 1 – common carbonate stone, sample 2 – CH marble, submitted to dry biocleaning (DB) and not treated (C – control), were analyzed and the results are reported in **Table 1**.

#### Physical aspects

The color changes measured at 48 hours, at laboratory-scale after dry biocleaning with *S. cerevisiae* yeast cells, are reported in **Table 1**. The partial color differences ΔL*, Δa*, Δb* and total color difference between two samples ΔE*_ab_ values were calculated. Comparing color values before and after dry biocleaning, on the surface fragment sample 1 - common carbonate stone (**Figures 1A** and **B**), no significant differences were recorded. In fact, in this case, the lightness changed only slightly from its original ΔE*_ab_ value of 12.60. This response is important since the trial was performed in order to test, for the first time, the application of de-hydrated yeasts cells on a common stone surface where the spontaneous re-hydratation occurred at room temperature. In this case, the fragment stone adopted was a sound rock without any surface alteration due to the exposition to eventual pollutants of the environment; so, we did not expect any changes.

On the contrary, when the dry biocleaning (DB) with *S. cerevisiae* yeast cells was performed comparing the cleaned CH marble fragment (2) *versus* the untreated altered marble surface (C-Control) at 48 hours, remarkable differences in lightness were recorded. In fact, the partial color differences, ΔL*, Δa*, Δb* and total color difference ΔE*_ab_ between two samples (DB vs. C) were calculated with the ΔL* lightness values of 49.30 and the ΔE*_ab_ value of 49.80.

Finally, in parallel tests, color measurements on the untreated altered surface of marble stone fragment (C-Control) were compared to the primitive marble fragment artwork (C vs. fresh cut), like preliminary color level reference between altered or not, surface marble condition. Changes in color parameters in ancient marble fragment (sample 2) had a total ΔE*_ab_ of 4.40, where the partial color differences ΔL* had a value of 4.10; the Δa* and Δb* values were less of 5.

The capillary water absorption coefficient before and after the cleaned fragment surface (**Figures 1 A-F**) areas was evaluated by the contact sponge method. Few changes in water absorption coefficients were recorded by comparing high-quality stone fragments, before and after the biocleaning process, such as an increase of water uptake of 0.6 g min cm^2^.

#### Chemical aspects

The pH values (**Table 1**), measured on the stone surface layers under the treatment, showed changes from neutral values (6.9) to lower values of 6.1 and 6.3 recorded on A, common carbonate stone, and B, CH marble, respectively, at the end of the dry biocleaning process (T 48 hours). No variation was detected on the surface of C, the control sample.

Analytical techniques were adopted to detect isopropyl alcohol, the organic solvent, on the stone surface as a catabolic intermediate of initial anaerobic fermentation by inoculated yeasts. The results showed the presence of alcohol at the end of the treatment (48 hours), at very low levels, confirming metabolic yeast processes.

#### Biological aspects

The biological parameters monitored before and after the biocleaning process at the laboratory scale are shown in **Table 1**. The data of the total viable bacterial and fungal counts determined on stones surfaces of common carbonate stone (1), CH marble (2) and the control (C), showed no statistical changes when comparing before and after the process evidencing low count values. On the contrary, when dry biocleaning using dried *S. cerevisiae* cells occurred, the yeast count increased on the surface of artwork confirming the favorable re-hydration conditions. The intense metabolic microbial activity recorded was further confirmed by the relevant content of ATP on the stone surface submitted to biocleaning, compared to the values determined in the control areas without yeast.

The enzymatic profiles of *S. cerevisae* yeast cells at the end of dry biocleaning treatments (48 hours), evaluated by Apy-Zym test kit, are reported in **Table 1**. The carbon source mixed to the dried yeast cells, added as inoculum in the biocleaning process, affected the total number of enzymes involved in the biochemical metabolic activities, with eleven and nine, present on the common carbonate stone and CH marble, respectively, whereas only two enzymes were present on the control.

### On-site dry biocleaning trials at The Four Fountains, Rome, Italy

**Figure 2:**
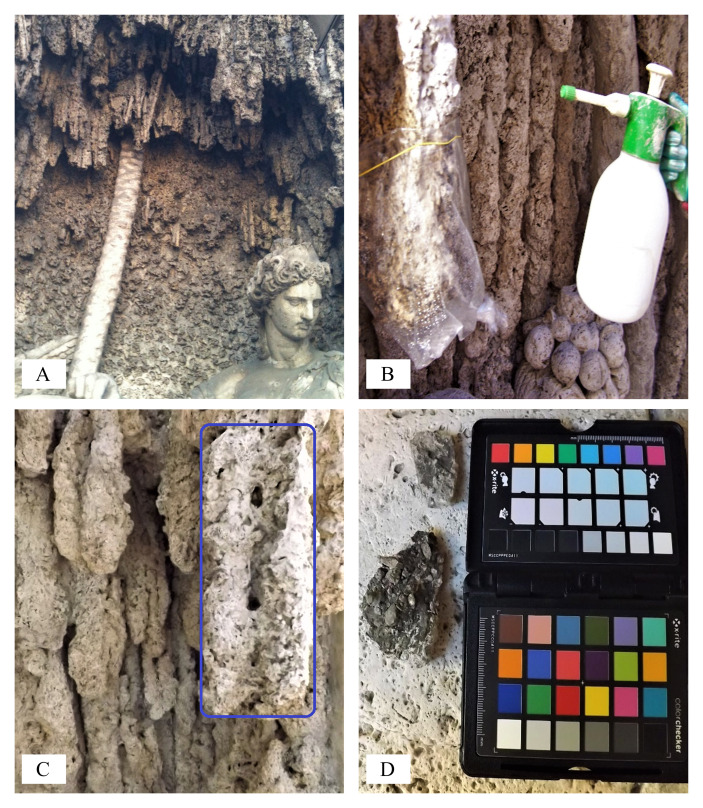
FIGURE 2: Views of the fountain depicting *Juno* during the biocleaning process. Detail of the dark-ridge representative of the fountain ornaments **(A)**; detail of the dry biocleaning process on travertine material ornaments before and after **(B, C)** the dry cleaning technique, and color scale by digital camera and ColorChecker X-Rite **(D)**.

The area selected for the on-site dry biocleaning process was part of one of *The Four Fountains* which is dedicated to *Juno*. IC analyses of the main chemical composition and SD of the travertine stone materials from the selected area (1,000 cm^2^), at T0 and at T18 (hours), including nitrate, sulfates, chloride, phosphate, sodium, magnesium and calcium ions, and comparing two different treatments (dry biocleaning (DB) and nebulization (N)), are reported in **Table 2** and **Figure 2**.

The on-site dry biocleaning system at *The Four Fountains* was optimized as follows:

-) characterization of parameters at time zero (see “Materials and Methods”);
-) manual bioapplication of dried yeasts onto the travertine stone surface;
-) use of a thin plastic film to cover the area treated by dry biocleaning;
-) gentle cleaning of the surface by soft sponge, left to dry in the outdoor environment;
-) final characterization of the same parameters as in the first point.

**TABLE 2.** **On-site physical-chemical and biological characterization on *The Four Fountains* surface after the dry biocleaning process (DB), compared to mechanical treatment by nebulization (N) and untreated control (C).** The amounts of nitrate, sulfate, chloride, sodium, magnesium, calcium and potassium ions determined by IC analyses are reported in % w/w (three replicates, mean ± SD).

**Parameters**	**On-site treatments at *The Four Fountains***	**Dry Biocleaning (DB)**	**Nebulization(N)**	**Control (C)**
	**Time (h)**	18	18	0
Physical	Water absorption (g)	3.2±0.4	3.4±0.5	1.8±0.4
	Color changes	DB *vs.* C	N *vs*. C	C *vs*. fresh cut
	ΔL*	40.9	42.30	49.40
	Δa*	0.17	−1.42	−1.45
	Δb*	10.1	−0.18	0.55
	ΔE*_ab_	42.20	42.55	49.90
Chemical	pH	6.4±0.1	6.9±0.2	7.1±0.2
	Alcohol (%)	0.30±0.1	0.00±0.1	0.00±0.1
	Nitrate (NO_3_^−^)	0.16±0.01	0.28±0.02	1.85±0.95
	Sulfate (SO_4_^2−^)	2.20±0.40	2.65±0.30	10.60±1.7
	Chloride (Cl^−^)	0.03±0.01	0.03±0.01	0.38±0.10
	Sodium (Na^+^)	0.08±0.02	0.16±0.05	0.27±0.08
	Magnesium (Mg^2+^)	0.12±0.06	0.14±0.07	0.35±0.09
	Calcium (Ca^2+^)	0.42±0.02	2.23±0.08	4.41±0.20
	Potassium (K^+^)	0.05±0.01	0.07±0.01	0.18±0.02
Biological	Bacteria (TVBC) (CFU cm^−2^)	1±2	1±1	4±2
	Fungi (CFU cm^−2^)	3±2	1±2	5±2
	Yeasts (CFU cm^−2^)	19±3	1±1	1±1
	ATP (pg cm^−2^)	3,200±5.5	48±8	65±15
	Number of enzymes	8	1	3

#### Physical aspects

The color change calculated at 18 hours, at onsite after dry biocleaning with *S. cerevisiae* yeast cells, are reported in **Table 2**. The partial color differences ΔL*, Δa*, Δb* and total color difference between two samples ΔE*_ab_ values were calculated. The total color changes at 18 hours, at on-site dry biocleaning (DB) by *S. cerevisiae* yeast cells led to a remarkable difference between the lightness of the cleaned and the untreated altered surface (control, C). Before and after the dry biocleaning treatment (DB) the total lightness changed to a final ΔE*_ab_ value of 42.20, getting a close value of the polished stone, by water nebulization (N) which is 42.55. The comparison of the untreated altered travertine stone (C) with both the dry biocleaned (DB) and the mechanical cleaning surfaces by water nebulization (N) indicated that the partial color differences for Δa* were always less than or equal to 1.5, whereas Δb* and ΔL* was, in the first case, greater than 10 and, in the latter case, 40.9. In our study, *E* was largely due to the variation in L*(lightness) and b* (difference between yellow and blue), while a* (difference between green and red), was considerably smaller.

Furthermore, comparing the partial and total color differences among the altered untreated travertine surface (control, C) to the freshly cut surface for travertine lithotype, the lightness showed a ΔL* value of 49.40 and a final ΔE*_ab_ value of 49.90, indicating a great level of altered travertine surface exposed at outdoor environmental conditions.

The L* chromatic differences between both the dry biocleaning (DB) - water nebulization (N) and control (C) tests exceed the threshold of 5 CIELAB units, indicating that not only an experienced observer could notice the difference [61, 62].

The results of the capillarity water absorption tests carried out on the travertine surface of the selected biocleaned areas for both the nebulization and control tests showed the absence of significant changes between the first two treatments. The heavily ornamented surface of the artwork may have affected the variability of the measured capillarity data. In such cases, perhaps the adoption of mini-tests, with smaller diameter sponges, might be more appropriate.

Environmental temperatures during the on-site experimental test at *The Four Fountains* ranged from 11°C to 22°C (night and day), while air humidity ranged from 75% to 90%.

**Figure 3:**
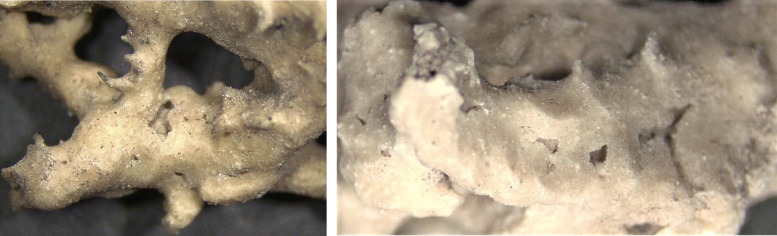
FIGURE 3: Stereomicroscope observations (20 x) of travertine material ornaments on CH *Four Fontains* fragment before (a-left) and after (b-right) the dry biocleaning technique (Photos credits to P. Di Marzio).

Stereo-microscope observations (20x) of travertine material ornaments on the *Four Fountains* fragment before and after the dry biocleaning technique are report in **Figure 3**. Micrographs show dark material irregularly distributed on the sample surface before the dry biocleaning treatment; after the biocleaning it is possible to notice a sensible reduction of gray - brown deposit, a de-coloration effect on the surface, without marked changes or alteration on the stone materials.

#### Chemical aspects

The pH, measured directly on the surfaces being treated, showed changes from values near neutral (6.9-7.0) to lower values of 6.4 recorded at the end of the dry biocleaning process (18 hours) (**Table 2**). The slight acidification, related to fermentation caused by the yeast's metabolism when the cells grow on substrates rich in hydrocarbon sources, was confirmed by detection of isopropyl alcohol, the organic solvent, on on-site stone surfaces. The very low levels of alcohol present at the end of the treatment (18 hours) were useful to confirm an initial fermentative activity by the yeasts.

All the samples related to the on-site *Four Fountains* surfaces (dry biocleaning (DB); nebulization (N) and control (C) tests, respectively), were also analyzed by means of IC and reported as % w/w (**Table 2**).

Control replicate samples (C 1-3) of stone powder from the surface showed high contamination by soluble salts (presumably gypsum), such as nitrate, sulfate, calcium and magnesium; instead, the amounts of ions, such as chloride, sodium and potassium were lower.

After dry biocleaning, the mean of all three sample replicates (DB 1-3) contained lower residual amounts of all salts determined than the control samples (C 1-3): nitrate, chloride and calcium were present in lower amounts (less than 10%), followed by sulphate, sodium, potassium and magnesium in higher amounts (residual contents of about 25-40% compared to the C 1-3 samples from the control area).

n N1-3 samples the amounts of residual salts on the surfaces after nebulization treatment were lower than the control (C), but higher than the dry biocleaning process (DB); in fact, the mean reduction values were nitrate (15%), sulfate and chloride (30%), potassium, magnesium and calcium (35-50%) and sodium (60%).

**Figure 4:**
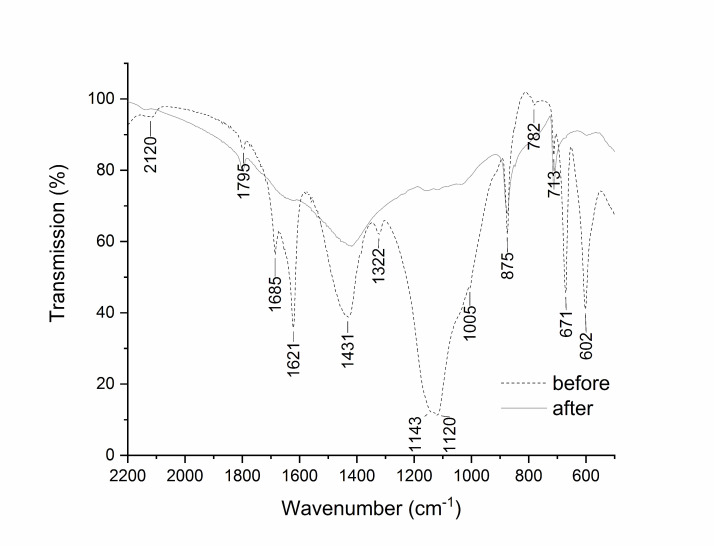
FIGURE 4: FTIR transmittance analysis of a representative area of fragment B before (dotted line) and after (solid line) the biocleaning. The spectrum recorded before the treatment (dotted line), shows the signals of calcium carbonate (1795, 1431, 875, 713 cm^−1^), gypsum (1685, 1621, 1143, 1120, 671, 602 cm^−1^) and calcium oxalate (1322, 782 cm^−1^).

The FTIR spectra of the surface before the treatment (**Figure 4**) showed peaks at 1431 cm^−1^ (asymmetric C=O stretching), 875 cm^−1^ (out-of-plane bending vibration) and 713 cm^−1^ (in-plane bending vibration), that are the typical absorbances of calcium carbonate (CaCO_3_) from the carbonate substrate [63, 64]. The peaks at 1685 and 1622 cm^−1^ and the bands at 1143 cm^−1^ and 1120 cm^−1^ could be ascribed to hydroxyl bending vibrations and SO asymmetric stretching, respectively, suggesting the presence of gypsum (CaSO_4_ 2H_2_O) [53]. The signals at 671 and 602 cm^−1^ (SO asymmetric bending modes) are confirmatory. The bands at 1322 and 782 resemble the specific signal of C=O stretching and OCO bending vibrations ascribed to calcium oxalate (CaC_2_O_4_ *n*H_2_O) [65]. The intensity of the signals due to gypsum and calcium oxalate decrease noticeably in the spectrum recorded at the end of the biocleaning treatment (48 hours).

The decreasing trend was confirmed by IC, which is more sensitive than FTIR spectroscopy, and still reveals the presence of sulphate mostly related to gypsum, together with fluoride, nitrate, and chlorine ions in the water extraction fraction of the powder, while nitrite, bromide and phosphate ions were below the detectable limit. The weight of the markers per unit area decreased after the marble surface was biocleaned, in particular with regard to fluorine and sulphate ions that decreased from 0.191 to 0.003 mg cm^−2^ and from 0.524 to 0.030 mg cm^−2^, respectively.

#### Biological aspects

Microbiological parameters used to monitor the biocleaning process at *The Four Fountains*, are reported in **Table 2**. The total viable bacteria count (TVBC) and fungi determined on the travertine surface submitted to dry biocleaning and nebulization areas were low, and no significant differences were found compared to the control. The yeast counts on the travertine surface submitted to dry biocleaning showed significant higher values (*p* <0.05), (more than 10 times – 19 CFU cm^−2^) both compared to the nebulization (N) and the control (C) areas.

**Figure 5:**
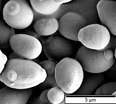
FIGURE 5: SEM image of *S. cerevisiae* yeast cells at the end of dry biocleaning process (18 hours) at on-site *The Four Fountains*.

The dry biocleaning system, where dried *S. cerevisiae* cells were added together with powdered sucrose directly onto the surface of the fine artwork, was effective within the time of treatment of 18 hours, indicating that, under favorable re-hydration conditions, the yeast cells have a remarkable cleaning capability in terms of salt reduction and pollutant removal at the environmental temperatures recorded. When dry yeasts cells have been sprayed onto the artwork surface, in few minutes the air humidity leads to spontaneous re-hydration inducing a change in the metabolic activity of the yeast cells. This was confirmed by repeated and direct optical microscope and SEM observations of sterile cotton swabs from the surfaces of the artwork submitted to biocleaning. The morphology of the dried yeast cells, added as inoculum, showed quick variation in hydration state, increasing in volume and cell turgidity (**Figure 5**).

Additionally, micro-bubbles of gas formation, indicating yeast respiration processes, were noted.

Under the on-site environmental conditions, the ATP contents measured on the travertine stone surfaces increased in value only where dried yeasts were added as inoculum in the biocleaning process as shown in **Table 2**.

The ATP content, as a bioindicator of the overall metabolic activities of the yeast, increased from 200 minutes on until 12-14 hours, reaching a total ATP value of 3,200 pg cm^−2^. The data agree with the total viable microbial counts indicating that these results are of great interest for methods that employ yeast inocula (for type, carbon source and amount, time of biotreatment, temperature, set up and optimization of the biological protocol, etc.).

**Figure 6:**
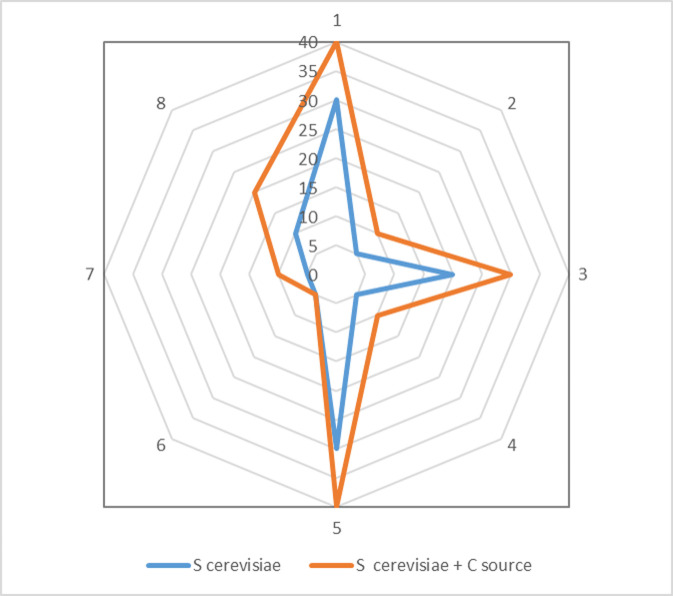
FIGURE 6: Enzymatic profiles of *S. cerevisiae* yeast cells at the end of dry biocleaning process (18 hours) on-site at *The Four Fountains*, in the presence and absence of a carbon source (Mean of two replicates). Key: 1. Alkaline phosphatase; 2. Esterase (C4); 3. Leucine arylamidase; 4. Valine arylamidase; 5. Acid phosphatase; 6. Phosphoamidase;7. α-Glucosidase; 8. α-Mannosidase.

Data reported in **Table 2** show that the enzymatic profiles of *S. cerevisae* yeast cells had changed at the end of the dry biocleaning treatments, at 18 hours, indicating that some metabolic activities had occurred; indeed, the carbon source added at time zero affected the kinetics of the reaction, expressed in nanomoles of substrates hydrolyzed, in seven of the eight cases. However, it did not affect the diversity, in terms of the total number of enzymes (19) detectable in the biochemical test response adopted in our experimental conditions are shown in **Figure 6**.

Short and medium-term (at one and three months, respectively) monitoring of the effects of the biocleaning process showed no significant differences in the treated area compared to the control areas, both for the total viable microbial counts (bacteria, yeasts and fungi), that remained <log 1.0 CFU cm^−2^, and for the stone-surface color changes.

### Economic evaluation of the dry biocleaning restoration

An economic evaluation of the dry biocleaning includes several factors, like source of microbes, growth of microbial biomass, practical operations for the application and conservation, duration treatment, monitoring, removal, effectiveness and safety toward artworks, people and the environment.

A preliminary cost-analysis of the dry biocleaning process showed that the use of commercial dried yeast cells is less expensive than the use of bacterial isolates, considering both the work required to isolate pure cultures and the cost of a specific bacterial strain after reactivation in culture media. In fact, the total cost can be quantified as being approximately 25-30 € for 1.0 kg of dried yeast and 3-5 € for the pure micronised powdered sucrose, using ½ kg of the yeast-sucrose suspension for about 20 m^2^ of artwork surface. On the basis of the feedback obtained from the professional conservation scientists involved in this study and from the comparison of the costs of the dry biocleaning process to the conventional mechanical and/or chemical-physical techniques (when compatible with the nature and ornamentation of artwork), it appears that the latter may be less convenient. However, on this matter, further evaluation will be considered taking several factors into account like the time and the number of applications required for restoration and the costs linked to specialized personnel, restorers and environmental safety.

## DISCUSSION

The preliminary lab-scale results obtained using the dry biocleaning, and subsequently, the on-site treatment on outdoor artwork, based on spontaneous re-hydratation process, can be both considered positive and promising. To our knowledge, for the first time selected dried yeast has been considered and used in biocleaning altered and fragile works of art; in particular, when the other traditional techniques could be dangerous or not applicable, active yeast cells showed to represent an innovative and good alternative solution as a bio-restoration method in the CH field.

In this context, the use of eukaryotic yeast cells, such as *S. cerevisiae*, instead of prokaryotic bacteria, can offer certain advantages in our opinion. A single vital cell can be compared to an industry that bases its activity on a natural balance between catabolic and anabolic processes, i.e. a metabolism that is beneficial for the organism. A different size of organisms (from smaller to greater), usually corresponds to both a correlation with the complexity of their life in nature, and the ability to offer several advantages. If we compare the average dimension and ATP content of a *Escherichia coli* cell (size 1.0 um and 1.0 pg/ATP) to a *S. cerevisiae* yeast cell, the last show size of 100 nm and ATP content until 200 pg/ATP [13]. Even if used in technological processes and/or microbial fermentations, they express a different number of proteins and enzymes involved, with often very different metabolic rates and production yields. Furthermore, *S. cerevisiae* is well known to be involved in alcohol fermentation of several organic substrates containing carbohydrates like grape musts for wine and malt extract for beers [66–68].

In the food industry, yeasts are best known for their beneficial role in the production of fermented products and bread, even if we cannot exclude biofilm formation on food-processing equipment and food-contact materials like source of microbial contamination [69].

On the base of the preliminary positive results, although achieved on a limited area of *The Four Fountains* complex, both the Superintendency and the Technical Commission for Restoration (Roma Capitale, Italy) approved to extend, in future biocleaning intervention, the use of *S. cerevisiae* in pure culture to treat a larger or even total surface area of another fountain and artworks affected by similar alterations.

For the first time, a novel dry biocleaning process is reported and represents an important pilot case-study confirming the positive role played by the microbial biotechnologies in the CH field. It represents a new opportunity for the research area in the CH bio-recovery. In addition, the results discussed in this paper, showed that the dry biocleaning process is efficient at removing alterations on stone surfaces, and that it has a low impact, it is easy to use, inexpensive and can be considered a practical alternative to any conventional treatments.

It may also be an opportunity to make the biotreatment even less invasive by considering the possibility of reducing or eliminating the addition of a carbohydrate source along with the yeast cell starter. This will make the final cleaning and any risk of microbial contamination over time safer.

To date, a number of appropriate microorganisms have been already used in dry biocleaning for the remediation and recovery of altered CH stonework affected by atmospheric pollutants, including nitrogen and sulfur oxides which are compounds that rapidly accelerate the deterioration of artwork and carboxylic acids, that could promote the formation of calcium oxalate films [64]. In the last few decades, biocleaning treatments have provided promising, innovative solutions for CH conservation, but the development and application of both new products and advanced protocols, raises concerns regarding risks for the safety of artwork, people and the environment [70, 71]. Trying to respond to these concerns, we must consider sustainable approaches, implementing multidisciplinary studies for all sectors involved in preserving CH. Starting from an original idea, or a problem, or the needs of the key operators in the CH sector to support products and/or protocols, the developers must provide safe bio-formulates and procedures for conservation, ensuring their efficiency and effectiveness. Furthermore, assessment of sustainability must include the comparison of traditional, consolidated procedures with innovative methods and strategies.

In conclusion, this study gives a new scientific contribution to the promotion and use of safe microbes, like *S. cerevisiae* cells yeast in CH biorestoration field. The obtained results, both at lab and onsite scale, although on small area, show the potential of the dry biocleaning as gentle and innovative biotechnology, able to offer the recovery of delicate and degraded artworks. For the first time, it represents an important result towards a new potential microbial protocol for CH recovery. The findings discussed in the paper, show that the dry biocleaning process is efficient at removing alterations on delicate stone surfaces, and that it is sustainable having a low environmental impact, easy to use, and safe. The process uses selected commercial dried safe yeasts, their spontaneous re-hydratation under environmental conditions and the related metabolic activity.

However, additional investigations are needed on several and more extensive alterations, the overall results on the development of an innovative and promising strategy will lead to further discussion and applications in the CH biotechnological field.

We believe that, by optimizing the parameters in relation to the different characteristics of the works of art to be treated, the biological process based on dry biocleaning could offer practical and economic advantages. However, the application of the method in wider cases would require: (i) constant interdisciplinary involvement of people with expertise in CH recovery (scientists and conservators); (ii) a great number of case studies on altered artwork; (iii) a rapid, accurate method to monitor biological activity and to avoid any undesirable effects and possible risks; (iv) the correct, complete removal of residual biological activity; (v) the standardization of the process in order to establish a clear, fast biotreatment protocol.

## MATERIALS AND METHODS

### Object and site description

The laboratory scale trial was carried out on two stone blocks to assay the pioneeristic dry biocleaning (DB) process: a common carbonate stone from the Campitello Matese area (sample 1), and an ancient marble fragment from OpaPisa, Pisa, laboratories (sample 2) [54].

The on-site application was performed at the “*Quattro Fontane*” (*The Four Fountains*), a complex of fountains from the twelfth century, installed under the pontificate of Pope Sisto V (1347-1358) and commissioned to decorate the streets of the *Colle del Quirinale (Quirinale Hill)*. The fountains, erected in the characteristic wall-type style, all consist of a semicircular travertine basin leaning against a niche that encloses a statue lying on its side with a marshy background. Travertine is a limestone rock of chemical origin formed in the continental environment by the evaporation of spring waters or limestone rivers. Calcium carbonate deposited by incrustation on shrubs and leaves decayed to putrefaction, creating a characteristic porous, vacuolar structure in the rock, called “*spugnone*”, that is typically an off-white, almost yellowish color.

**Figure 7:**
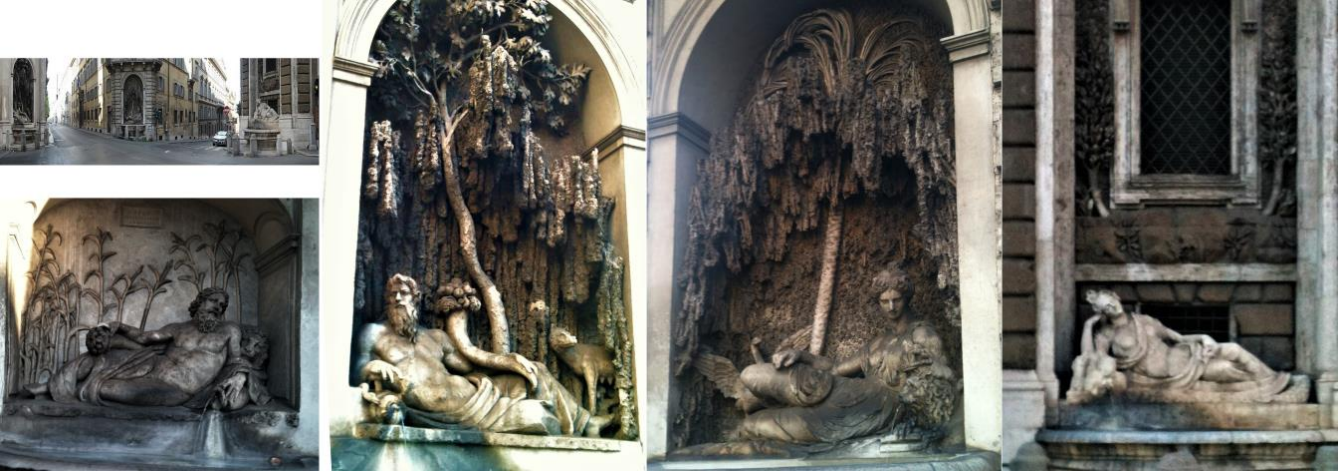
FIGURE 7: View of the “*Quattro Fontane*” (*The Four Fountains*) complex, in Rome (Italy).

The “*Quattro Fontane*” is located at a nexus of Roman city traffic in an area that, additionally, is particularly exposed to variable weather conditions, to very high levels of gaseous pollutants, soot-particles and dust from vehicular traffic (**Figure 7**). The fountains were in a great decayed state characterized by the presence of large deposits and incrustations with dark brown chromatic alterations. Numerous restorations of *The Four Fountains* have been performed over the years and the last radical interventions took place in 1936. Previous hygroscopic studies on one fountain have revealed high soluble salt content combined with the high porosity of travertine materials, humidity and the high level of air pollution, resulting in a darkened appearance [72].

### Sampling and material characterization

#### Samples

Micro-samples were taken with a steel scalpel both from the specimens under laboratory testing and from *The Four Fountains* artwork on site, in a way that was as little invasive as possible. The specimens, chosen from the bases of their representative geometric forms, were a fragment from a common carbonate stone with quite a flat surface (thickness 2.0 cm; about 150 cm^2^) and a CH fragment of marble from a historic campestris statue with a curved surface (height 14 cm; diameter base, 10 cm) as shown in **Figure 1**.

Three representative areas (1.0 cm x 1.0 cm) of the marble surface were scraped by a scalpel before and after biocleaning. The powders were finely ground in a mortar and analyzed with chemical techniques.

#### Infrared spectroscopy

Micro-samples taken from the specimens used in the laboratory tests were scraped and the powder was analyzed by Fourier transform infrared (FTIR) spectroscopy in order to characterize the composition of the surface and the variation in compounds present, depending on the intensities of their associated peaks [73, 74]. FTIR spectra were recorded from KBr pellets (Sigma-Aldrich FTIR Grade) in transmission mode by a BioRad Excalibur Series FTS 3000 spectrometer (detector DTGS) in the 4000–400 cm^−1^ range with a resolution of 4 cm^−1^ [63, 75].

#### Ionic chromatography

Anion and cation content from water-soluble salts was determined by ion chromatography [53]. Samples for the extraction of soluble salts were treated according to the UNI NORMAL method. Ten mg were weighed for every sample, then 10 mL of ultra-pure water (Milli-Q Gradient A10 system, Millipore - 18MΩ resistivity, <5 ppb TOC) were added. The resulting solutions were exposed to mechanical agitation at 300 rpm for 17 hours, by means of a Stuart SSL1 orbital shaker. The solutions were filtered with disposable 0.45 µm filters and then diluted. For cation analysis, the samples were acidified to pH 3.0 with ultra-pure HNO_3_ produced by sub-boiling distillation (DuoPUR unit, Milestone).

Ionic chromatography analysis was performed by means of a Metrohm IC Compact 761, equipped with a Metrohm 831 Compact Autosampler. Anions were analyzed using a Metrosep A Supp 5 240/4.0 column, 0.7 mL min, 13 Mpa, 20 µL injection volume, eluent Na_2_CO_3_ 1.8 mmol L^−1^ - NaHCO_3_ 1.7 mmol L^−1^. Cation analysis was performed by means of a Metrosep C2 150/4.0 column, 0.9 mL min, 6.8 Mpa, 20 µL injection volume, eluent H_3_PO_4_ 5 mmol L^−1^.

### Biocleaning agents and culture methods

A pure culture of dried yeast *S. cerevisiae* var. *bayanus* cells (Garzanti Specialties SpA, Milan, Italy) was used as biocleaning agent in the dry biocleaning tests (DB) and compared to a mechanical cleaning system using vapor nebulization (N). Dried yeast cells (*SB aroma* type) were stored at 4-8 °C under vacuum in 500 g pockets.

The yeast strain was preliminarily tested in order to evaluate total yeast count (TYC expressed as CFU g^−1^) and purity; incubation was at 28°C for 24-36 hours, on a Yeast Peptone Dextrose Agar (YPDA) medium (Oxoid Ltd., Basingstoke, UK). Further, enzymatic activity was evaluated by quali-quantitative enzyme profiles (Apy-Zym test kit, BioMerieux, Florence, Italy) and microbial growth was monitored by OD_560_ and ATP content [76]. The strain was stored on YPDA medium (Oxoid), at 4 °C.

### Dry biocleaning procedure under laboratory conditions and on-site

#### Lab experiments

Aliquots of dried yeast cells at approximately 10^9^ CFU g^−1^ were added to a dried carbohydrate source as powdered sucrose (1/10, w/w), and applied directly onto the stone surface with a manual spray device on top of the artwork. After yeast application, the stone fragments were placed in a 3 L jar (GasPak system, BD BBL, USA), with 10 mL of sterile tap water on the cotton layer at the bottom, avoiding direct contact with the stone. This was then closed and stored in an incubator at 28°C to ensure the correct metabolic activity of the dried yeast.

After 48 hours, the stone fragments were submitted to gentle hand-cleaning by soft sponge, dampened with sterile de-ionised water. The stone surfaces were dried at room temperature (22°C, 48 hours) and submitted to analyses.

#### On-site experiments

Bio-applications, aliquots of dried yeast cells and sucrose were stored separately at 4°C and mixed just before use. In order to protect the stone surface and to create a separate mini-environment able to induce a moisture condensation phenomenon, after the application of the yeast-sucrose mixture with a manual spray device on the artwork, a single layer of plastic PET film (Domopak, Volpiano, Italy) was applied to the surface.

The experimental design on the entire altered *Four Fountains* artwork surfaces consisted of:

- dry biocleaning (DB) on-site treatments on a small representative area of about 1,000 cm^2^, with contact times ranging from 12 to 18 hours;
- nebulization treatment (N) as the main cleaning process adopted for the overall area of the artwork. The nebulizer used was Grifo model nebulizer and spray nozzle delivers water at 1.33 L min^−1^ (Phase Restauro srl, Firenze, Italy) droplets range 80-120 µm for 18 hours
- control areas (C), where no treatments were applied (untreated).

At the end of the process, the treated and control areas were submitted to three gentle manual cleanings, using a soft sponge soaked in sterile water, and dried at room temperature or under outdoor conditions, according to Ranalli *et al.* [52].

### Monitoring of the effectiveness of the dry biocleaning process

The different aspects considered in the process are reported as follows:

#### Microbes

One very important aspect of any cleaning and biocleaning treatment is the monitoring of the effectiveness of the process. After the final drying step, in order to check the possible presence of residual yeast cells and/or microbial contaminants (bacteria and fungi), sterile swabs (Fissan, Milan, Italy) were used to ensure that no treatment residues were left on the artwork surfaces; the data were compared with analyses carried out before the treatments. Microbiological counts were performed using Potato Dextrose Agar for Micromycetes, such as yeast and fungi (YPD Agar, Oxoid), and Standard Plate Count Agar for aerobic heterotrophic bacteria (SPCA, Oxoid).

#### ATP

Moreover, the ATP content was simultaneously determined using a specific enzymatic kit (NRM/Lumit-Qm; Lumac B.V., Landgraaf, The Netherlands). A Biocounter 1500 P luminometer (Lumac B.V.) equipped with a photomultiplier tube set at 7,200 RLU with 200 pg ATP in 100 uL of Lumit buffer and Lumit-QM reagent was used [77].

#### Enzymes

Enzymatic activities of yeast cells were determined by the API-ZYM system (bio-Merieux Italia, Rome, Italy). Semi-quantitative evaluation of the activities of 19 hydrolytic enzymes (alkaline phosphatase, esterase (C4), esterase–lipase (C8), lipase (C14), leucine arylamidase, valine arylamidase, cistine arylamidase, trypsin, a-chymotripsin, acid phosphatase, phosphoamidase, a-galactosidase, b-galactosidase, b-glucuronidase, a-glucosidase, b-glucosidase, N-acetil-b-glucosamidase, a-mannosidase and a-fucosidase) were determined using the API-ZYM system [78]. Two replicates were always performed for the dry biocleaning and the control tests. The reproducibility was >95%.

During the tests, if conditions allowed it, pH (PH200 mod. portable meter, Thomas Scientific, UK; pH-strip and Quantofix Relax, Velp Scientifica, srl, Italy) and alcohol formation measurements were performed by a digital alcohol test analyzer, range sensitivity 0.00-1.99 g L^−1^ (TechaBit mod., Buckles Ltd., Barking, Essex, UK).

#### Capillarity

Capillarity water absorption tests were carried out to estimate the water uptake coefficient on the travertine surface of selected areas and controls using the sponge contact method, a non-destructive on-site methodology [79]. The UNI Normal procedure requires use of a sponge with a known density (Spontex type Calypso) soaked in water, weighed, and placed on the surface of the material for one minute under a given pressure, then re-weighed [80].

#### Temperature and moisture

These parameters were constantly monitored both in the laboratory and on-site by portable data loggers (RHiLog Escort Data Loggin System Ltd., Auckland, New Zealand).

#### Color measurements

Miniscan with light D65 (Hunter Lab, Bergamo, Italy) was adopted in order to evaluate the efficiency of the biotreatment. The chromatic coordinates L* (lightness axis moves from a top value of 100=white to a bottom value of 0=black), a* (axis is associated with changes between red and green), and b* (axis is associated with changes between yellow and blue) of the stone surfaces were determined [81, 82]. According to the CIELAB 1976 system, the partial color differences ΔL*, Δa*_,_ Δb* and total color difference between two samples ΔE*_ab_ [(L*)^2^ (a*)^2^ (b*)^2^]^1/2^, were calculated. The ΔE*_ab_ >5 is perceived by the human eye [29, 61, 83].

Color measurements on dry biocleaned (after, DB) vs. altered surface (before, control, C), and on cleaned surface by nebulization (after, N) vs. altered surface (before, control, C) were carried out. In addition, color measurements on freshly cut surface for each lithotype treatment (marble and travertine fragments) *vs* altered surface (control, C) were calculated.

#### Microscopic observations

Laboratory and portable models of optical OM - Nikon Eclipse E600 model (Nikon Instruments Europe B.V. Amsterdam, Netherlands) and stereo SM-Zeiss AxioScope (Carl Zeiss Spa, Milan, Italy) microscopy, connected to high-resolution digital cameras, were adopted. Fragments of collected stone samples and cotton swabs obtained at pre-set intervals from the surfaces of the artwork submitted to biocleaning were examined.

Furthermore, the yeast rehydration and the growth cell during the biocleaning treatment were observed using scanning electron microscopy (SEM). The samples for SEM observation were left to sit overnight in a solution of 2% glutaraldehyde (0.01 mol L^−1^ phosphate buffer), and then immersed in 1% osmium tetroxide. A microscope operating at 10 kV was used (Zeiss DSM 940A; LEO Elektronenmikroskopie GMbH, Oberkochen, Germany).

### Statistical analysis

Experiments (physical-chemical, microbial count and biochemical data) were expressed as mean ± standard deviation (SD) and statistical analysis between tests was calculated by a Chi-square test using SPSS 11.5 (SPSS for Windows, USA); the significance level was set at *p* < 0.05.

## Abbreviations:

CH: – Cultural Heritage.
